# Deletion of GPR81 activates CREB/Smad7 pathway and alleviates liver fibrosis in mice

**DOI:** 10.1186/s10020-024-00867-y

**Published:** 2024-07-09

**Authors:** Ying Zhi, Kerui Fan, Shuang Liu, Kai Hu, Xinyan Zan, Ling Lin, Yongqiang Yang, Xianqiong Gong, Kun Chen, Li Tang, Longjiang Li, Jiayi Huang, Shujun Zhang, Li Zhang

**Affiliations:** 1 Department of Pathophysiology, Basic Medical College, Chongqing Medical University, 1 Yixueyuan Road, Chongqing, 400016 China; 2 Laboratory of Stem Cell and Tissue Engineering, Chongqing Medical University, Chongqing, China; 3 Department of Anatomy, Basic Medical College, Chongqing Medical University, Chongqing, China; 4 Hepatology Center, Xiamen Hospital of Traditional Chinese Medicine, Xiamen, Fujian China; 5 Chongqing Key Laboratory of Infectious Diseases and Parasitic Diseases, Department of Infectious Diseases, The First Affiliated Hospital of Chongqing Medical University, Chongqing, China; 6 Laboratory of Integrated Traditional and Western Medicine, Chongqing Traditional Chinese Medicine Hospital, Chongqing, 400011 China

**Keywords:** G-protein-coupled receptor 81, Liver fibrosis, cAMP, CREB, Smad7

## Abstract

**Background:**

Enhanced glycolysis is a crucial metabolic event that drives the development of liver fibrosis, but the molecular mechanisms have not been fully understood. Lactate is the endproduct of glycolysis, which has recently been identified as a bioactive metabolite binding to G-protein-coupled receptor 81 (GPR81). We then questioned whether GPR81 is implicated in the development of liver fibrosis.

**Methods:**

The level of GPR81 was determined in mice with carbon tetrachloride (CCl_4_)-induced liver fibrosis and in transforming growth factor beta 1 (TGF-β1)-activated hepatic stellate cells (HSCs) LX-2. To investigate the significance of GPR81 in liver fibrosis, wild-type (WT) and GPR81 knockout (KO) mice were exposed to CCl_4_, and then the degree of liver fibrosis was determined. In addition, the GPR81 agonist 3,5-dihydroxybenzoic acid (DHBA) was supplemented in CCl_4_-challenged mice and TGF-β1-activated LX-2 cells to further investigate the pathological roles of GPR81 on HSCs activation.

**Results:**

CCl_4_ exposure or TGF-β1 stimulation significantly upregulated the expression of GPR81, while deletion of GPR81 alleviated CCl_4_-induced elevation of aminotransferase, production of pro-inflammatory cytokines, and deposition of collagen. Consistently, the production of TGF-β1, the expression of alpha-smooth muscle actin (α-SMA) and collagen I (COL1A1), as well as the elevation of hydroxyproline were suppressed in GPR81 deficient mice. Supplementation with DHBA enhanced CCl_4_-induced liver fibrogenesis in WT mice but not in GPR81 KO mice. DHBA also promoted TGF-β1-induced LX-2 activation. Mechanistically, GPR81 suppressed cAMP/CREB and then inhibited the expression of Smad7, a negative regulator of Smad3, which resulted in increased phosphorylation of Smad3 and enhanced activation of HSCs.

**Conclusion:**

GPR81 might be a detrimental factor that promotes the development of liver fibrosis by regulating CREB/Smad7 pathway.

**Supplementary Information:**

The online version contains supplementary material available at 10.1186/s10020-024-00867-y.

## Introduction

A growing body of evidence suggests that metabolic reprogramming is a critical pathological event driving the progression of various disorders, including liver fibrosis, a common consequence of chronic hepatic disorders(Gilgenkrantz et al. 2021; Trivedi et al. 2021). Enhanced glycolysis has been regarded as a typical feature of liver fibrosis(Wang et al. 2024), while inhibition of glycolysis results in beneficial outcomes in experimental animals with liver fibrosis(Xiang et al. 2023; Li et al. 2023). However, the molecular mechanisms underlying the detrimental significance of glycolysis in fibrogenesis have not been fully understood.

Lactate is the endproduct of glycolysis, which has long been well known as a waste product or a metabolic intermediate that supplies gluconeogenesis(Li et al. 2022). Interestingly, recent studies have found that lactate functions as a ligand of G-protein-coupled receptor 81 (GPR81), a Gi-coupled receptor also known as hydroxycarboxylic acid receptor 1 (HCAR1) (Li et al. 2022). The ligation of GPR81 by lactate or other agonists, such as 3,5-dihydroxybenzoic acid (DHBA), suppressed cyclic adenosine monophosphate (cAMP)/protein kinase A (PKA)/cAMP-responsive element-binding protein (CREB) pathway and involved in the regulation of lipolysis, inflammation, and tumorigenesis(Ahmed et al. 2010; Vardjan et al. 2018; Brown et al. 2020; Khatib-Massalha et al. 2020). Thus, lactate functions as a signaling molecule with profound bioactivities.

Several studies have found that GPR81 is critical for the development of inflammation-based disorders, such as sepsis and colitis(Yang et al. 2022; Ranganathan et al. 2018), but the potential roles of GPR81 in liver fibrosis remains unclear. In the present study, the pathological significance of GPR81 in liver fibrosis was investigated in GPR81 deficient mice with carbon tetrachloride (CCl_4_)-induced liver fibrosis, a widely used animal model of liver fibrosis(Otto et al. 2023; Xu et al. 2023). In addition, the GPR81 agonist DHBA was administered in CCl_4_-challenged mice to verify the experimental results in GPR81-deficient mice. To the best of our knowledge, this is the first study that revealed the detrimental roles of GPR81 in liver fibrosis.

## Materials and methods

### Materials

CCl_4_ was the product of Chengdu Kelong Chemical Reagent Factory (Chengdu, China). The recombinant human TGF-β1 was purchased from PeproTech (#100-21, Suzhou, China). The alanine aminotransferase (ALT, #C009-2-1), aspartate aminotransferase (AST, #C010-2-1), and hydroxyproline (HYP, #A030-2-1) assay kits were obtained from Nanjing Jiancheng Bioengineering Institute (Nanjing, China). SYBR Green (#AG 11701), Steady PureQucik RNA Extraction Kit (#AG 21023), and EVO M-MLV Reverse Transcriptase (#AG11605) were obtained from Accurate Biotechnology (Changsha, China). The Cyclic adenosine monophosphate ELISA Kit (cAMP, #E-EL-0056c) was produced by Elabscience Biotechnology (Wuhan, China). The primary antibodies used for immunoblot analysis in this study included: GPR81 (#A20321), TGF-β1 (#A16640), alpha-smooth muscle actin (α-SMA, #A17910), collagen I (COL1A1, #A16891), phosphorylated Smad3 (#AP0727), Smad3 (#A19115), and Smad7 (#A12343) were purchased from ABclonal Technology (Wuhan, China). Phosphorylated CREB (#AF5785) and CREB (#AF6566) were purchased from Beyotime Institute of Biotechnology (Jiangsu, China). The β-actin antibody was purchased from 4A BIOTECH (#ICM001-100, Beijing, China). HRP-linked goat anti-rabbit and goat anti-mouse antibodies were purchased from CST (#7074, Danvers, MA, United States). The BCA protein assay kit (#23225) and the enhanced chemiluminescence (ECL) reagents (#WP20005) were purchased from Thermo Fisher Scientific (Rockford, IL, USA).

### Animals

Male C57BL/6J mice (6 weeks, 16–18g) were obtained from the Experimental Animal Center of Chongqing Medical University. *GPR81*^+/-^ mice on a C57BL/6J background were purchased from Cyagen Biosciences Inc. (Guangzhou, China) and self-mated to generate *GPR81*^*−/−*^ homozygous mice (GPR81 KO) and wild-type control mice. The primer sequences used for genotype identification are listed in Table 1. All mice were housed under specific pathogen-free conditions with controlled temperature and a 12 h light/dark cycle. After all treatment, mice were anesthetized with 5% chloral hydrate and sacrificed. All animal experiments in this study were performed in accordance with and approved by the Ethics Committee of Chongqing Medical University.

**Table 1:** The primer sequences used for genotype identification

Gene	Forward primers (5′-3′)	Reverse primers (5′-3′)
GPR81-knockout	TTTCTAATACCGGGCGGATGTTTC	GGCCACCTGGATGTTCAAACCT
GPR81-wild-type	TTTCTAATACCGGGCGGATGTTTC	ACCAGGATGAGTAGAGGAGGCATCA

### CCl_4_-induced liver fibrosis

Liver fibrosis was induced in mice by intraperitoneal injection of CCl_4_ (1 ml/kg, dissolved in olive oil) or the vehicle (olive oil) twice per week, for 8 weeks. To determine the significance of GPR81, CCl_4_-challenged mice received once-daily administration of GPR81 agonist DHBA (30 mg/kg, dissolved in normal saline) or the vehicle (saline). Three days after the last injection of CCl_4_, the mice were anesthetized with 5% chloral hydrate and sacrificed. The left lobe of the liver and the blood sample were harvested for further experiments.

### Histological examination

Liver tissues were fixed in 4% paraformaldehyde, dehydrated, embedded in paraffin, and sliced into 4 μm-thick sections. To determine the histological abnormalities, the sections were stained with hematoxylin and eosin (H&E). To reveal the degree of collagen deposition, liver sections were subjected to Masson's trichrome staining and Sirius red staining using commercial kits from Solarbio (Beijing, China) according to the manufacturer's instructions. The positive area and total dyed area were examined using Image Pro Plus 6.0 software by measuring five randomly chosen, non-overlapping fields at a magnification of 100 ×, and the average positive area was calculated. The results were represented as a fold change ratio toward the relative control group.

### Cell culture and treatment

The human hepatic stellate cell line (LX-2) was purchased from National Collection of Authenticated Cell Cultures (Shanghai, China) and cultured using Dulbecco’s Modified Eagle’s Medium (DMEM) high glucose (#11995065, Gibco) supplemented with 5% (v/v) FBS and 1% (v/v) Penicillin–Streptomycin solution and was maintained at 37 ºC in a humidified atmosphere containing 5% CO_2_. LX-2 is an immortalized human hepatic stellate cell line prepared by Xu et al. through SV40 T-antigen transformation in 2005. The mycoplasma, bacterial, and fungal tests were performed on the cells before use, and the results were negative.

LX-2 cells were exposed to FBS-free medium overnight, and then the culture media was replaced by fresh media supplemented with recombinant human TGF-β1 (#100-21, PeproTech) at a concentration of 10 ng/ml or the vehicle. To determine the role of GPR81, DHBA was supplemented at a concentration of 1 mM. 24 h later, cells were collected for mRNA or protein extraction.

### Determination of biochemical parameters

To evaluate the degree of liver injury, the mice blood samples were collected and centrifuged (5000*g*, 4 °C, 15 min) to obtain serum for analysis. Serum ALT and AST were determined with the Alanine aminotransferase/Aspartate aminotransferase Assay Kit (Nanjing Jiancheng Biotech) according to the manufacturer’s instructions.

To evaluate the degree of liver fibrosis, the liver tissues were homogenized in PBS and then centrifuged at 8000*g* for 10 min to collect the supernatant for analysis. The hydroxyproline contents in liver tissues were determined with the Hydroxyproline Assay Kit (Nanjing Jiancheng Biotech) following the manufacturer's instructions.

### Determination of cAMP by ELISA

Liver tissues were homogenized and the supernatants were collected. The contents of cAMP in the supernatants were evaluated using the cAMP ELISA Kit (Elabscience Biotechnology, China) according to the manufacturer's instructions. The hepatic content of cAMP was normalized by the total protein concentration of the supernatant, and the data were expressed as the fold change relative to the respective control.

### Quantitative real-time PCR analysis (RT-qPCR)

Total RNA was isolated from mice liver samples by using a SteadyPure Quick RNA Extraction Kit (Accurate Biology) and reverse-transcribed to cDNA with a reverse transcriptase kit (Accurate Biology) following the manufacturer’s instruction. Then Real-time PCR was performed with a SYBR Green RT-PCR Kit (Accurate Biology). The primers for GPR81, TNF-α, IL-6, TGF-β1, α-SMA, COL1A1, and Smad7 were synthesized by Accurate Biology (Changsha, China) and their sequences are listed in Table 2. The relative mRNA levels of GPR81, TNF-α, IL-6, TGF-β1, and Smad7 were normalized by that of β-Actin and were expressed as a fold change relative to the respective control.

**Table 2:** The primer sequences used for real-time PCR

Gene	Forward primers (5′-3′)	Reverse primers (5′-3′)
M-GPR81	GCGGAGGTCAGAAGAGATGC	CTCGTTGTTGGGCTGTTTGTC
H-GPR81	GCAGTCTGAAACCCAAGCAGC	GCCACACTGATGCAACTCCTG
M-TNF-α	CTGTCTACTGAACTTCGG	CCATAGAACTGATGAGAGG
M-IL-6	GGAGCCCACCAAGAACGAT	GTCACCAGCATCAGTCCCAA
M-TGF-β1	ATTTGGAGCCTGGACACACA	CGTAGTAGACGATGGGCAGT
M-α-SMA	GACAATGGCTCTGGGCTCTGTA	TTTGGCCCATTCCAACCATTA
H-α-SMA	AGAAGGAGATCACGGCCCTA	TGCTGGAAGGTGGACAGAGA
M-COL1A1	GTGTGTTCCCTACTCAGCCG	TGCTCTCTCCAAACCAGACG
H-COL1A1	TCGAGGGCCAAGACGAAGA	CGTTGTCGCAGACGCAGAT
M-Smad7	CTGCTGTGCAAAGTGTTCAGG	CCATTGGGTATCTGGAGTAAGGA
H-Smad7	ACTGTCCAGATGCTGTGCCTT	TATGCCACCACGCACCAGT
M-β-actin	CATCCGTAAAGACCTCTATGCCAAC	ATGGAGCCACCGATCCACA
H-β-actin	TGGCACCCAGCACAATGAA	CTAAGTCATAGTCCGCCTAGAAGCA

### Immunohistochemistry staining

Liver tissues fixated in paraffin were dewaxed, hydrated, treated with sodium citrate for antigen retrieval, and blocked with non-specific stain inhibitors (#KIT-9710, Maxim) for 10 min. Then, the sections were incubated with the primary antibody against GPR81 (#SAB1300792, Sigma-Aldrich) or α-SMA (#A17910, ABclonal) at 4 ℃ overnight, washed with PBS, and incubated at room temperature for 20 min with secondary antibodies. Next, the DAB developer was used for 1 min. Finally, sections were counterstained with hematoxylin for 10 min, dehydrated, and mounted. The positive area was quantified using Image Pro Plus 6.0.

### Immunoblotting

The liver samples were homogenized with RIPA lysis buffer to prepare tissue lysates. The total proteins were extracted after centrifuging for 15 min at 4 °C, and the concentrations were determined using BCA Protein Assay Kit (Thermo Fisher Scientific). The protein samples were subjected to SDS-PAGE and transferred to nitrocellulose membranes. After treating with blocking buffer (containing 5% skim milk, 10 mM Tris–HCl, 150 mM NaCl, and 0.1% Tween-20) for 1 h at room temperature, the membranes were incubated with the primary antibodies (the information about the antibodies is listed in Table 3) overnight at 4 °C and were incubated with the secondary antibody for 1 h at 37 °C. The bands were visualized with the enhanced chemiluminescence (ECL). Relative quantification of band intensities was carried out using ImageJ software (US National Institutes of Health). For the data shown, the gray value of the target protein was normalized with that of internal control, and the protein relative expression was represented as a fold change ratio toward the control group.

**Table 3:** Antibodies for immunoblotting and immunohistochemistry staining

Product	Company	Code	Dilution ratio
GPR81 Rabbit Polyclonal Antibody	Abclonal Technology	A20321	WB 1:1000
GPR81 Rabbit Polyclonal Antibody	Sigma-Aldrich	SAB1300792	IHC 1:100
TGF beta 1 Rabbit Polyclonal Antibody	Abclonal Technology	A16640	WB 1:1000
α-Smooth Muscle Actin (ACTA2) Rabbit Monoclonal Antibody	Abclonal Technology	A17910	WB 1:1000 IHC 1:100
Collagen I/COL1A1 Rabbit Polyclonal Antibody	Abclonal Technology	A16891	WB 1:1000
Phospho-CREB1 (Ser133) Rabbit Polyclonal Antibody	Beyotime Biotechnology	AF5785	WB 1:1000
CREB1 Rabbit Polyclonal Antibody	Beyotime Biotechnology	AF6566	WB 1:1000
Phospho-Smad3-S423/S425 Rabbit Monoclonal Antibody	Abclonal Technology	AP0727	WB 1:1000
Smad3 Rabbit Monoclonal Antibody	Abclonal Technology	A19115	WB 1:1000
Smad7 Rabbit Polyclonal Antibody	Abclonal Technology	A12343	WB 1:1000

### Statistical analysis

All data were shown as mean ± standard deviation (SD). For two group comparisons, an unpaired two-tailed Student´s t-test was used; for multiple comparisons, a one-way analysis of variance (ANOVA) followed by Bonferroni post hoc multiple comparison test was performed. Analyses were performed with GraphPad Prism (Version:8.4.2). The P < 0.05 was considered to be statistically significant.

## Results

### Chronic CCl_4_ exposure induced upregulation of GPR81

**Fig. 1:**
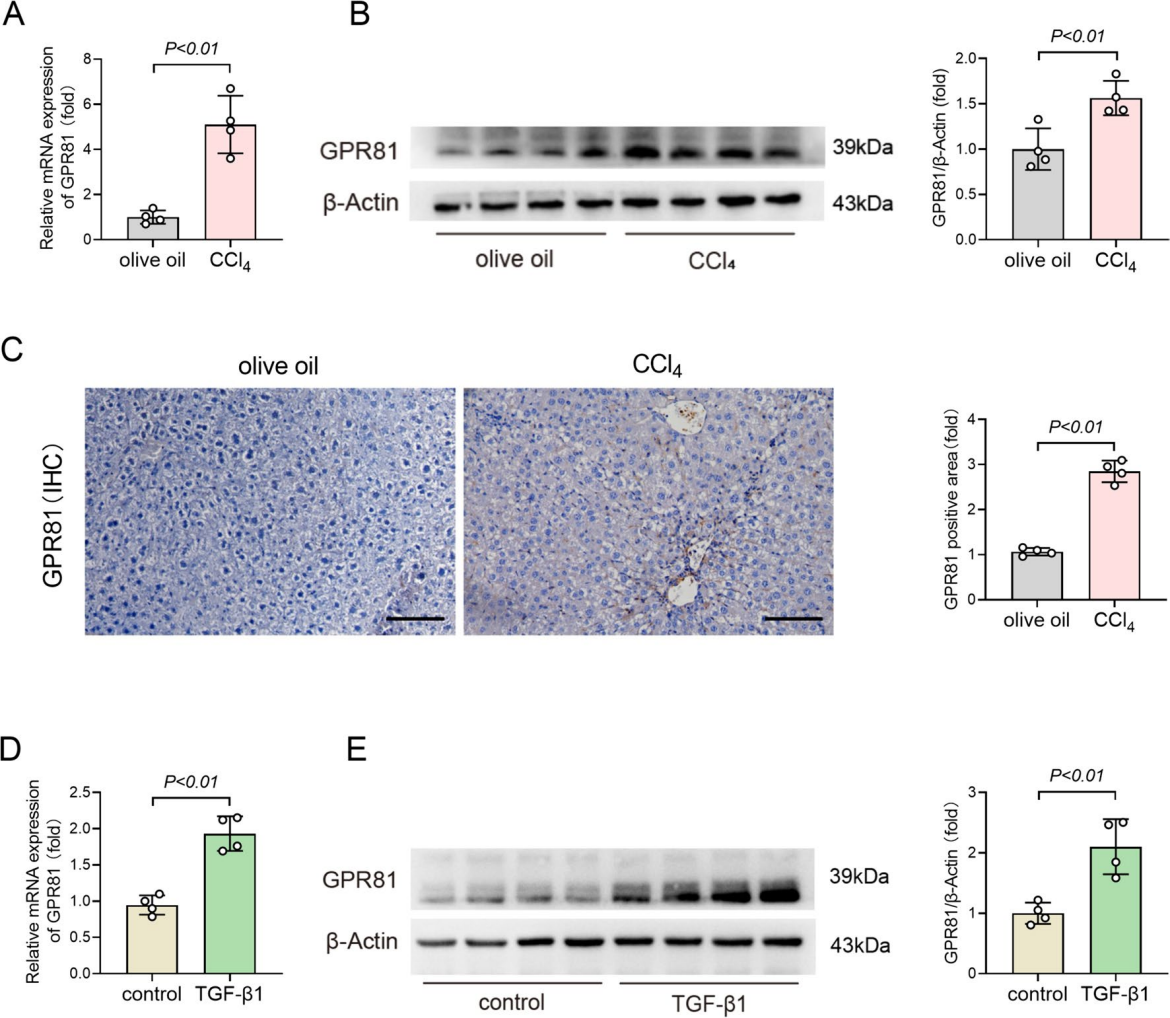
Chronic CCl_4_ exposure induced upregulation of GPR81. **A**–**C** The WT mice were intraperitoneally injected with olive oil or CCl_4_ for 8 weeks to induce liver fibrosis. **A** The hepatic mRNA and **B** protein expression of GPR81 were detected (n = 4). **C** Representative images of immunohistochemistry (IHC) staining of GPR81 (scale bar: 100 μm) were shown. The positive area was quantified and expressed as a fold change relative to the vehicle (olive oil) group (n = 4). **D**, **E** LX-2 human hepatic stellate cells were cultured and treated with vehicle or TGF-β1 to induce HSCs activation. **D** The mRNA and **E** protein expression of GPR81 were detected (n = 4). All data were expressed as mean ± SD

To investigate the potential involvement of GPR81 in the progression of liver fibrosis, hepatic levels of GPR81 in chronic CCl_4_-exposed mice were determined. The results indicated that chronic CCl_4_ exposure induced upregulation of GPR81 mRNA (Fig. 1A). Consistently, immunoblot analysis also found an increased expression of GPR81 in CCl_4_-insulted liver (Fig. 1B). Interestingly, immunohistochemical staining of GPR81 found that the upregulated expression of GPR81 was mainly distributed in periportal fields and along peripheral zone 1 (Fig. 1C), which is the typical region of collagen deposition in liver fibrosis (Lackner and Tiniakos 2019).

Since hepatic stellate cells (HSCs) play central roles in the overproduction of collagen (Friedman 2008; Iwaisako et al. 2014), we then questioned whether the activation of HSCs is associated with the upregulation of GPR81. As expected, the mRNA and protein levels of GPR81 were also up-regulated in activated LX-2 cells (Fig. 1D and E), a widely used human HSCs line in experimental studies (Pan et al. 2024; Fondevila et al. 2022). Interestingly, CCl_4_ and TGF-β1 also induced the elevation of lactate in liver and LX-2 cells (Supplementary Fig. 1A and B), respectively. The upregulation of both lactate and GPR81 suggests that GPR81 might be functionally involved in CCl_4_-induced liver fibrosis and HSCs.

### Deletion of GPR81 alleviated CCl_4_-induced chronic liver injury

**Fig. 2:**
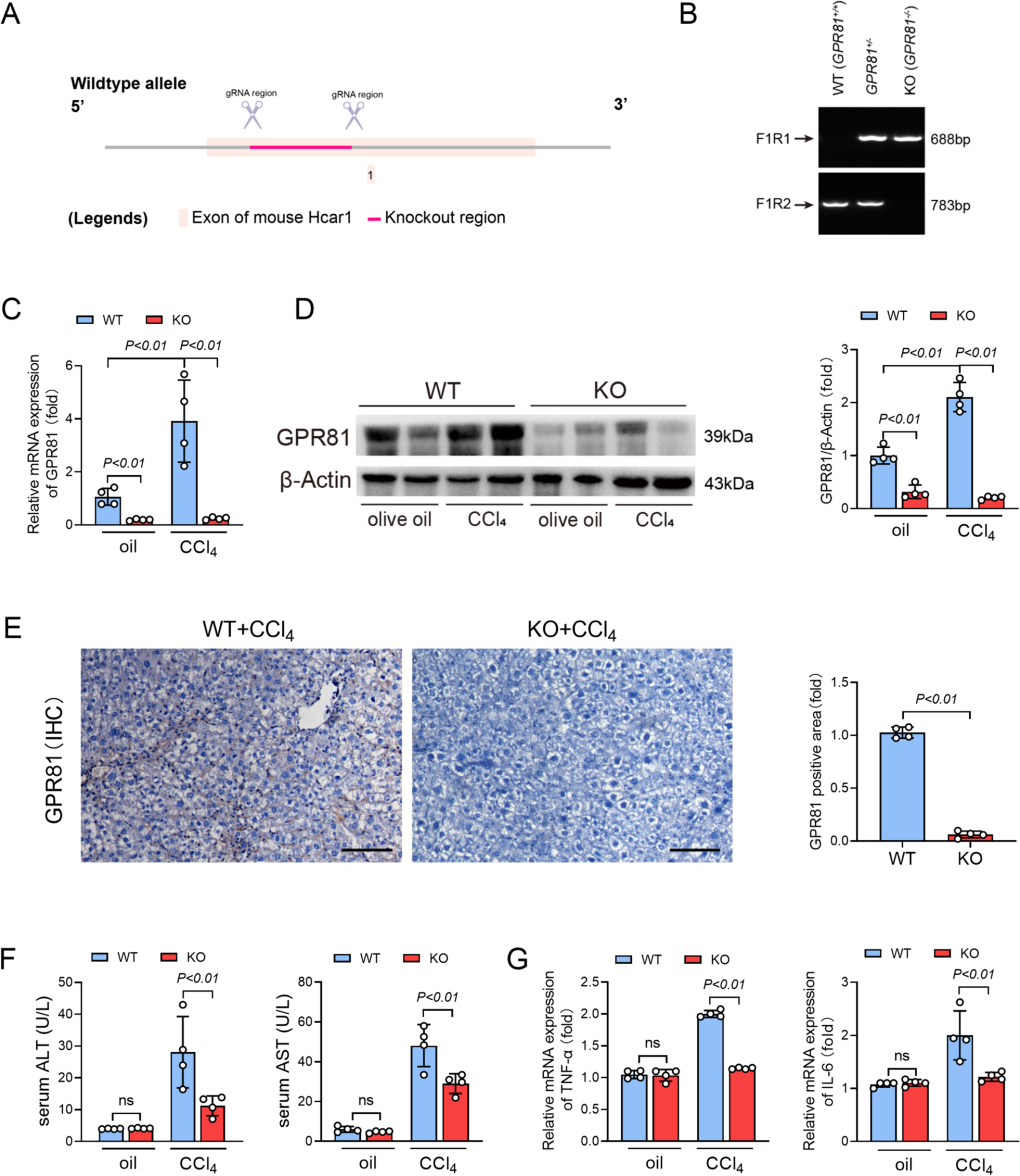
Deletion of GPR81 alleviated CCl_4_-induced chronic liver injury. The GPR81 KO mice were established and intraperitoneally injected with olive oil or CCl_4_ for 8 weeks to induce liver fibrosis. **A** Schematic representation of the strategy used to produce GPR81 KO mice. **B** The genotype of wild-type (WT) and GPR81 knockout (KO) mice. **C** The hepatic mRNA and **D** protein levels of GPR81 were detected (n = 4). **E** Representative images of immunohistochemistry (IHC) staining of GPR81 (scale bar: 100 μm) were shown. The positive area was quantified and expressed as a fold change relative to the WT group (n = 4). **F** The serum ALT and AST levels were examined (n = 4). **G** The mRNA levels of TNF-α and IL-6 were detected (n = 4). All data were expressed as mean ± SD

To investigate the potential roles of GPR81 in the development of liver fibrosis, GPR81 knockout (KO) mice were generated (Fig. 2A and B) and exposed to CCl_4_. As expected, CCl_4_-challenge failed to induce the upregulation of GPR81 in KO mice (Fig. 2C–E). As expected, no difference was detected in the olive oil-treated mice (Fig. 2F and G). However, deletion of GPR81 significantly suppressed CCl_4_-induced elevation of ALT and AST in serum (Fig. 2F). Deletion of GPR81 also resulted in alleviated morphological abnormalities of the liver, such as swollen and scattered patches (Supplementary Fig. 2). In addition, CCl_4_-induced upregulation of pro-inflammatory cytokines, including TNF-α and IL-6, were inhibited in GPR81 deficient mice (Fig. 2G). These results suggest that GPR81 might be a detrimental factor in CCl_4_-induced chronic liver injury.

### Deletion of GPR81 attenuated CCl_4_-induced fibrogenesis

**Fig. 3:**
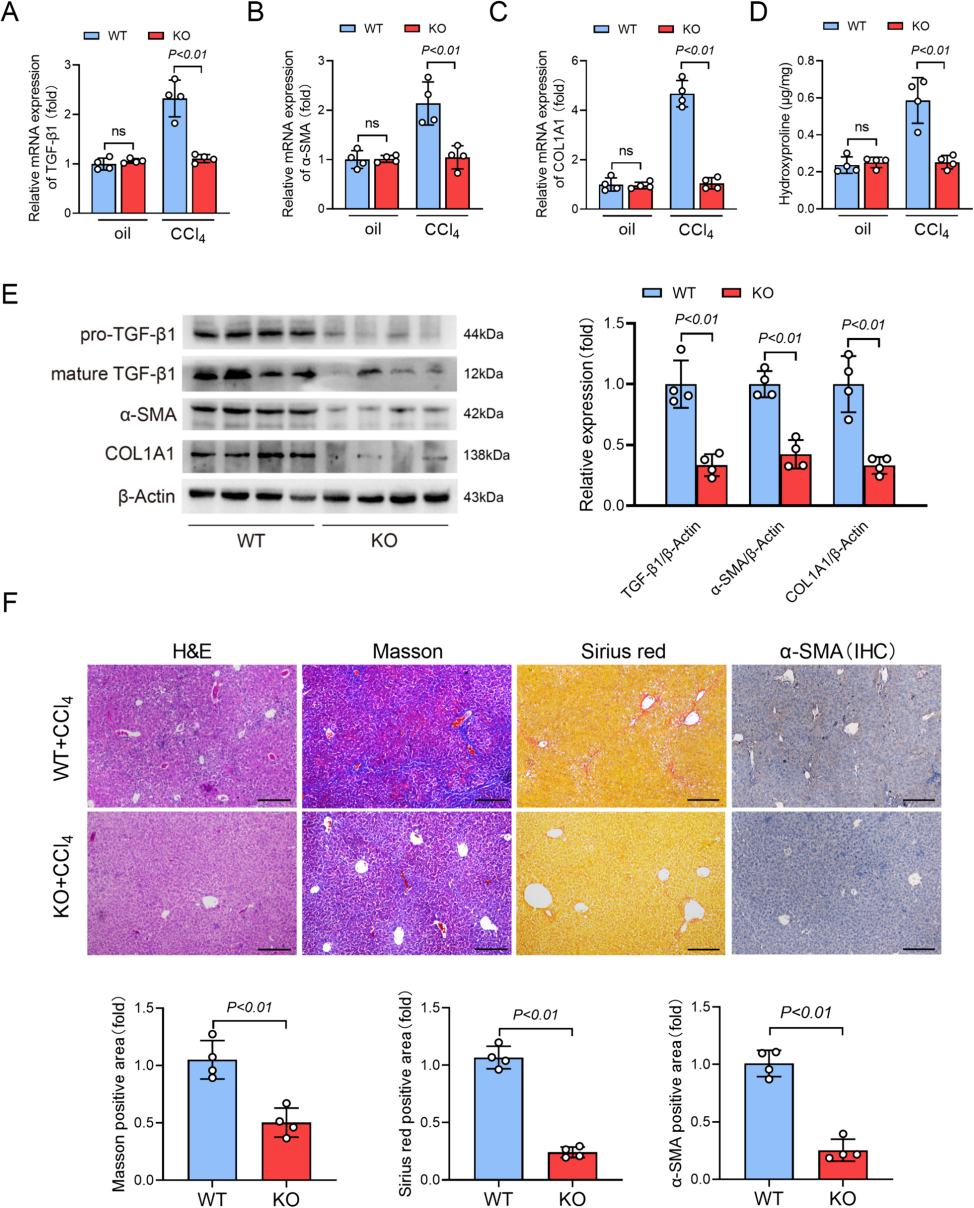
Deletion of GPR81 suppressed CCl_4_-induced fibrogenesis. WT mice and GPR81 KO mice were intraperitoneally injected with olive oil or CCl_4_ for 8 weeks to induce liver fibrosis. **A** The hepatic mRNA expressions of TGF-β1, **B** α-SMA, and **C** COL1A1 were examined (n = 4). **D** The hydroxyproline contents in liver tissues were detected (n = 4). **E** The protein levels of pro-TGF-β1, mature TGF-β1, α-SMA, and COL1A1 were examined (n = 4). **F** Representative images of H&E, Masson and Sirius red staining, and immunohistochemistry (IHC) staining of α-SMA of mice liver sections (scale bar: 50 μm) were shown. The positive area was quantified and expressed as a fold change relative to the WT group (n = 4). All data were expressed as mean ± SD

TGF-β1 is a central profibrogenic factor that plays an essential role in the activation of HSCs (Yang et al. 2023a). The upregulation of α-SMA is a molecular marker for the activation of HSCs, and the activated HSCs are the essential contributors to the synthesis of collagen and the deposition of extracellular matrix in liver fibrosis (Fan et al. 2022). The present study found no difference between olive oil-treated WT and KO mice, but the downregulation of TGF-β1, α-SMA, or COL1A1 in CCl_4_-treated GPR81 KO mice (Fig. 3A–C). In addition, deletion of GPR81 was not associated with the elevation of hydroxyproline (Fig. 3D), an amino acid specific to collagen (Udenfriend 1966). Thus, GPR81 deficiency does not induce spontaneous fibrogenesis in liver.

In mice that received repeatedly CCl_4_ challenge, the mRNA levels of TGF-β1, α-SMA, COL1A1, and the hepatic level of hydroxyproline increased significantly, which were all suppressed in GPR81 deficient mice (Fig. 3A–D). Consistently, immunoblot analysis found that the protein levels of pro-TGF-β1, mature TGF-β1, α-SMA, and COL1A1 were lower in CCl_4_-challenged GPR81 KO mice than those in CCl_4_-challenged WT mice (Fig. 3E). Additionally, the morphological examinations with H&E staining, Masson staining and Sirius red staining, and immunohistochemistry staining of α-SMA indicated that deletion of GPR81 alleviated liver injury, suppressed collagen deposition, and reduced α-SMA-positive area in CCl_4_-challenged mice (Fig. 3F). These results suggest that GPR81 might signal a profibrotic response in liver fibrosis.

### GPR81 activator aggravated CCl_4_-induced liver fibrosis

**Fig. 4:**
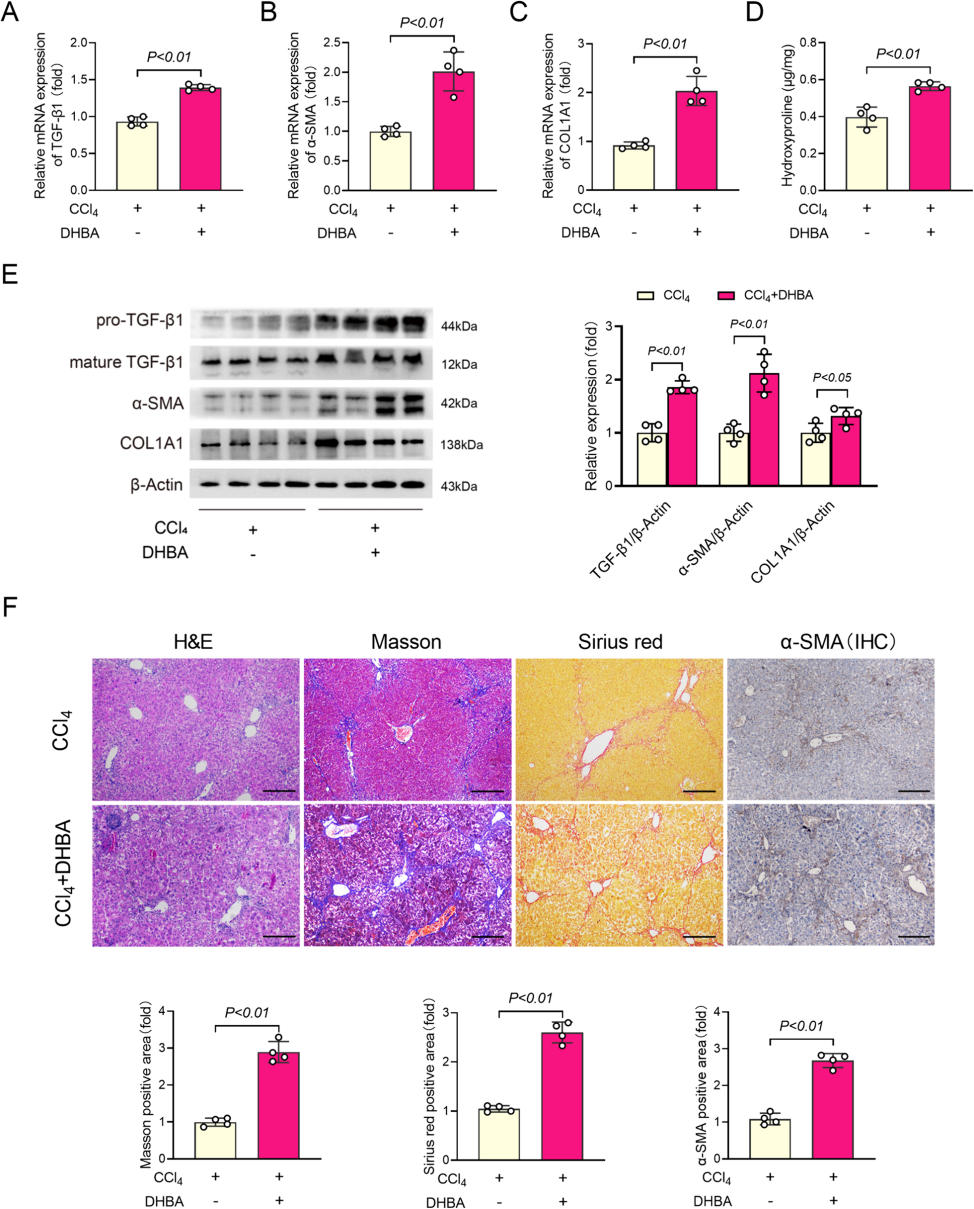
Treatment with GPR81 activator enhanced CCl_4_-induced fibrogenesis. WT mice with CCl_4_-induced liver fibrosis were supplemented with or without DHBA for 8 weeks. **A** The hepatic mRNA expressions of TGF-β1, **B** α-SMA, and **C** COL1A1 were examined (n = 4). **D** The hydroxyproline contents in liver tissues were detected (n = 4). **E** The protein levels of pro-TGF-β1, mature TGF-β1, α-SMA, and COL1A1 were examined (n = 4). **F** Representative images of H&E, Masson and Sirius red staining, and immunohistochemistry (IHC) staining of α-SMA of mice liver sections (scale bar: 50 μm) were shown. The positive area was quantified and expressed as a fold change relative to the CCl_4_ group (n = 4). All data were expressed as mean ± SD

DHBA is an activator of GPR81 which has been extensively used in experimental studies (Yili et al. 2023; Liu et al. 2012; Madaan et al. 2017). To validate the detrimental roles of GPR81 in the development of liver fibrosis, CCl_4_-exposed mice were treated with DHBA. In contrast to the result from GPR81 KO mice, supplementation with DHBA aggravated the morphological abnormalities of CCl_4_-insulted liver, increased the levels of ALT and AST in serum, and upregulated the induction of TNF-α and IL-6 in liver (Supplementary Fig. 3A–C), suggesting that CCl_4_-induced hepatic inflammation and liver injury were exacerbated by DHBA. In line with the aggravated liver injury, supplementation with DHBA increased the levels of TGF-β1, α-SMA, COL1A1, and hydroxyproline in CCl_4_-exposed mice (Fig. 4A–E). The histological examinations also found that supplementation with DHBA aggravated CCl_4_-induced histological lesions, enhanced CCl_4_-induced deposition of collagen, and increased α-SMA-positive area in CCl_4_-insulted liver (Fig. 4F). Interestingly, supplementation with DHBA failed to increase the level of ALT/AST, TNF-α/IL-6, or TGF-β1/α-SMA/COL1A1 in CCl_4_-challenged GPR81 KO mice (Supplementary Fig. 4A–C), suggesting that the detrimental effects of DHBA on CCl_4_-induced liver fibrosis depend on GPR81.

### GPR81 modulated CREB/Smad7 pathway

**Fig. 5:**
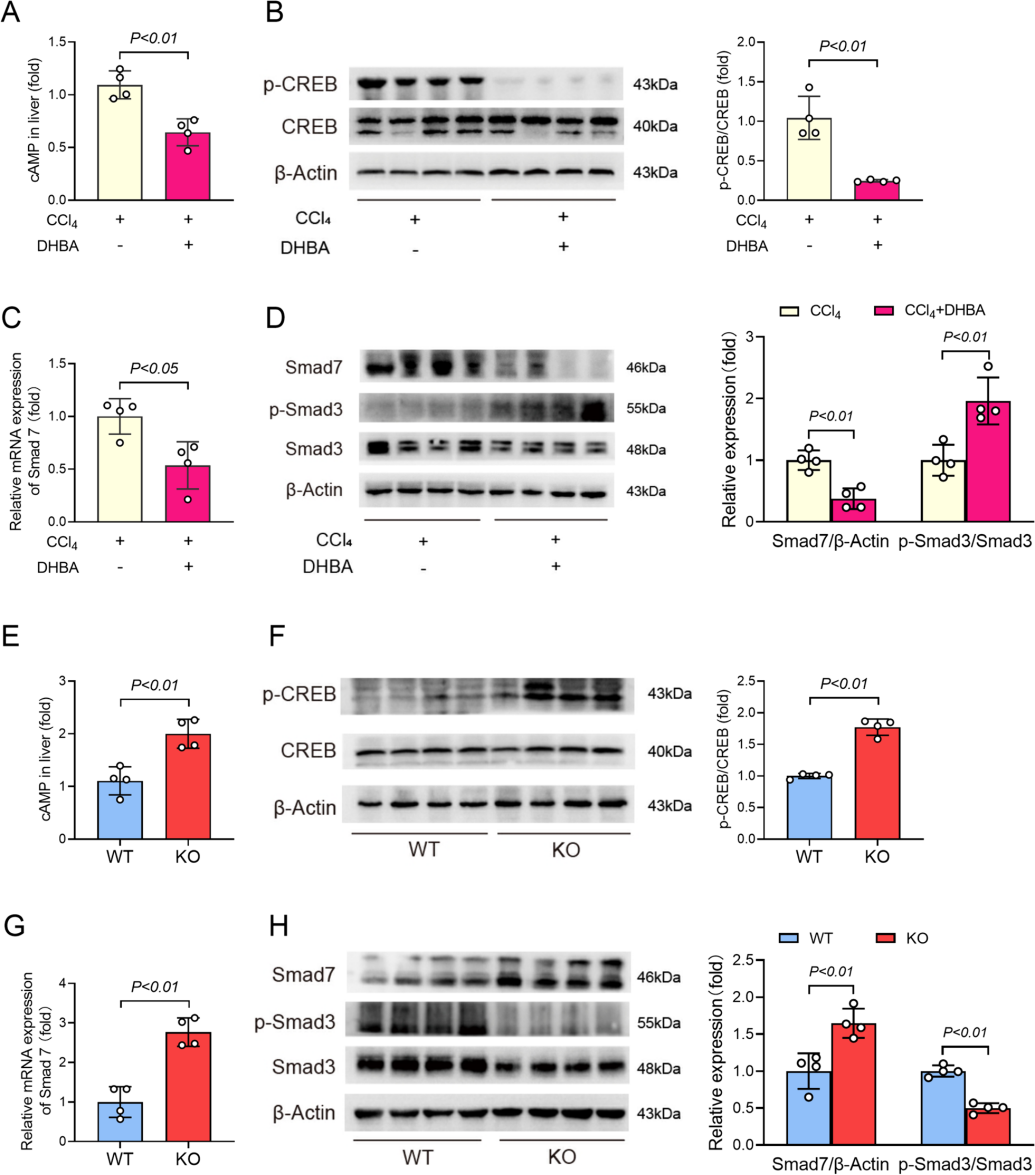
GPR81 modulated CREB/Smad7 pathway. **A**–**D** WT mice with CCl_4_-induced liver fibrosis were supplemented with or without DHBA for 8 weeks. **A** The hepatic contents of cAMP were detected by ELISA kit and were expressed as a fold change relative to the CCl_4_ group (n = 4). **B** The phosphorylation and total protein levels of CREB were determined (n = 4). **C** The mRNA levels of Smad7 were examined (n = 4). **D** The protein levels of Smad7, phosphorylated-Smad3 (p-Smad3), and total Smad3 (Smad3) were determined (n = 4). **E**–**H** WT mice and GPR81 KO mice were treated with CCl_4_ for 8 weeks to induce liver fibrosis. **E** The hepatic contents of cAMP were detected by ELISA kit and were expressed as a fold change relative to the WT group (n = 4). **F** The phosphorylation and total protein levels of CREB were determined (n = 4). **G** The mRNA levels of Smad7 were examined (n = 4). **H** The protein levels of Smad7, phosphorylated-Smad3 (p-Smad3), and total Smad3 (Smad3) were determined (n = 4). All data were expressed as mean ± SD

Several previous studies have found that GPR81 couples to Gi and activation of GPR81 reduces the level of cAMP (Ahmed et al. 2010; Khatib-Massalha et al. 2020), which functions as a major repressor of fibrotic responses (Delaunay et al. 2019). In agreement with these reports, the present study found that treatment with the GPR81 activator DHBA resulted in the reduction of cAMP (Fig. 5A). PKA/CREB pathway is essential for the anti-fibrotic activities of cAMP (Li et al. 2019). The present study found that treatment with DHBA also resulted in suppressed phosphorylation of CREB (Fig. 5B). Smad3 phosphorylation is an essential molecular event downstream of TGF-β1-induced fibrotic response (Zhang et al. 2023), but CREB-driven expression of Smad7 inhibits the phosphorylation of Smad3 (Gifford et al. 1979). Expectedly, supplementation with DHBA suppressed the expression of Smad7 but enhanced the phosphorylation of Smad3 (Fig. 5C and D). These results suggest that the detrimental effects of DHBA on liver fibrosis might be associated with suppressed CREB/Smad7 pathway.

On the contrary, the alleviated liver fibrosis in CCl_4_-insulted GPR81 deficient mice was associated with increased level of cAMP and enhanced phosphorylation of CREB (Fig. 5E and F). In addition, the hepatic level of Smad7 in GPR81 KO mice was higher than that in WT mice, while the hepatic level of phosphorylated Smad3 in GPR81 KO mice was lower than that in WT mice (Fig. 5G and H). These results further support that the pro-fibrotic activities of GPR81 might result from the inhibition of the anti-fibrotic CREB/Smad7 pathway.

### GPR81 activator suppressed CREB/Smad7 pathway in vitro

**Fig. 6:**
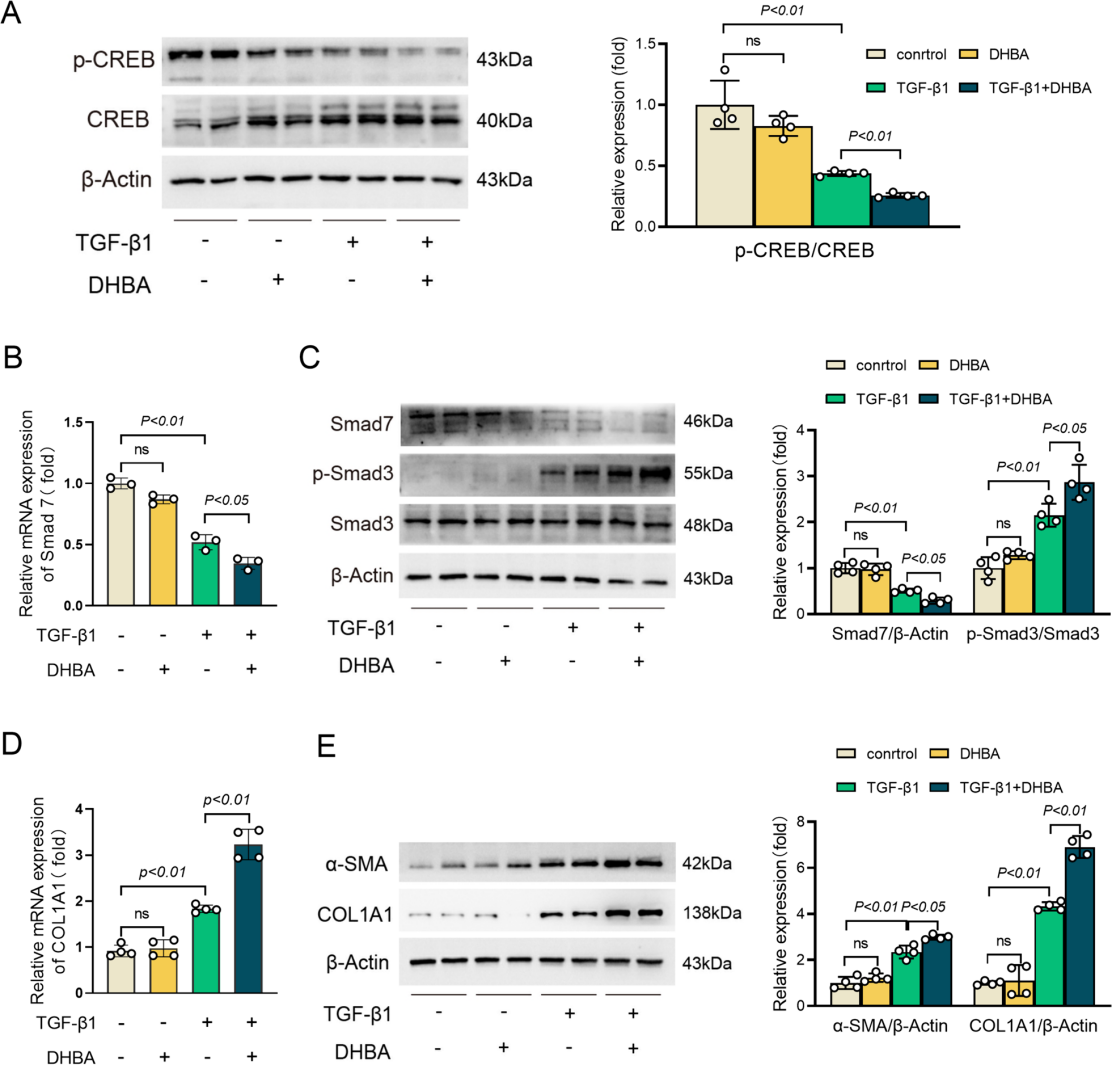
Treatment with GPR81 activator inhibited CREB/Smad7 pathway and promoted HSCs activation. Human hepatic stellate cells LX-2 with TGF-β1 exposure were supplemented with DHBA for 24 h. **A** The phosphorylation and total protein levels of CREB were determined (n = 4). **B** The mRNA levels of Smad7 were examined (n = 4). **C** The protein levels of Smad7, phosphorylated-Smad3 (p-Smad3), and total Smad3 (Smad3) were determined (n = 4). **D** The mRNA expressions of COL1A1 were examined (n = 4). **E** The protein levels of α-SMA and COL1A1 were examined (n = 4). All data were expressed as mean ± SD

To investigate the effect of GPR81 on HSCs activation, TGF-β1-exposed LX-2 cells were supplemented with the GPR81 activator DHBA. In line with the results in vivo, supplementation with DHBA suppressed TGF-β1-induced phosphorylation of CREB and expression of Smad7 (Fig. 6A–C) but enhanced TGF-β1-induced phosphorylation of Smad3 in LX-2 cells (Fig. 6C). Consistently, treatment with DHBA further increased TGF-β1-induced upregulation of α-SMA and COL1A1 in LX-2 cells (Fig. 6D and E). In addition, treatment with lactate also resulted in enhanced induction of α-SMA and COL1A1 in TGF-β1-activated LX-2 cells (Supplementary Fig. 5 A and B). These in vitro results further suggest that GPR81 might inhibit CREB/Smad7 pathway and then promote the activation of HSCs.

## Discussion

Accumulating evidence suggests that lactate, as well as other bioactive metabolites, is an essential regulator in signal transduction and pathological processes (Vardjan et al. 2018; Shang et al. 2022; Ryan et al. 2019). GPR81 has been identified as the receptor for lactate, and previous studies have found that GPR81 is involved in the development of colitis, sepsis, and cancer (Yang et al. 2022; Ranganathan et al. 2018; Ishihara et al. 2022). In the present study, CCl_4_-induced liver fibrosis is associated with upregulation of both lactate and GPR81, while deletion of GPR81 attenuated ECM deposition and alleviated histological abnormalities. Thus, GPR81 might be a detrimental factor that drives the development of liver fibrosis.

During the development of liver fibrosis, there is a notable metabolic shift from oxidative phosphorylation to glycolytic pathway (Delgado et al. 2021). It is well-known that the activation of macrophages, as well as other inflammatory cells, requires intensive metabolic support provided by enhanced glycolysis (Russo et al. 2021). Similar to this metabolic reprogramming in macrophages, the activation of HSCs is accompanied by an upregulated expression of several glycolytic enzymes (Zheng et al. 2020), and the present study also found that TGF-β1 induced the elevation of lactate in LX-2 cells. In addition, several other types of cells, including hepatocytes and endothelial cells, have been suggested to undergo a shift toward hyperglycolysis under the fibrotic conditions (Nishikawa et al. 2014; Inomata et al. 2022). Thus, the metabolic reprogramming in a variety of cells in fibrotic liver orchestrated the elevation of lactate.

Although the elevation of lactate has been suggested to result from the upregulation of glycolytic enzymes and enhanced glycolysis, the mechanisms involved in the transcriptional regulation of GPR81 largely remains unknown. A previous study has revealed the presence of functional peroxisome proliferator-activated receptor gamma (PPARγ)/retinoid X receptor (RXR) binding sites in the proximal promoter of the GPR81 gene (Jeninga et al. 2009). Under hypoxic conditions, hypoxia inducible factor-1 (HIF-1) binds to the promoter of GPR81 gene and stimulates the expression of GPR81 in lung mesenchymal progenitor cells (Yang et al. 2023b). In addition, the signal transducer and activator of transcription 3 (STAT3) has been found to directly bind to GPR81 promoter and activate GPR81 expression in lung cancer cells (Xie et al. 2020). Whether PPARγ, HIF-1, STAT3, or other unrevealed transcription factors are involved in the induction of GPR81 in liver fibrosis is worthy of further investigation.

In addition to using the GPR81 KO mice, DHBA, a widely used GPR81 agonist (Vohra et al. 2022; Ohno et al. 2018; Tassinari et al. 2024), was used in the present study to investigate the pathological significance of GPR81. A previous study suggested that the carboxyl group, the 3-hydroxyl group, and the 5-hydroxyl group of DHBA are crucial for the molecular interactions between DHBA and GPR81, and treatment with DHBA inhibited the release of free fatty acid and glycerol in adipocytes, but the antilipolytic effects of DHBA were abolished in GPR81-deficient adipocytes (Liu et al. 2012). In agreement with these findings, the present study found that supplementation with DHBA aggravated CCl_4_-induced liver fibrosis in WT mice but not in GPR81 KO mice. Thus, the pro-fibrotic effects of DHBA depend on GPR81, which also supports that upregulation/activation of GPR81 is a crucial molecular event driving the development of liver fibrosis.

Interestingly, immunohistochemical staining of GPR81 found that the upregulated expression of GPR81 was mainly distributed in the typical regions of collagen deposition in liver fibrosis (Goodman 2007; Kleiner et al. 2005; Gailhouste et al. 2010; Hadi et al. 2020). It is well-known that HSCs play central roles in the overproduction of collagen in the fibrotic region of hepatic lobule (Friedman 2008; Iwaisako et al. 2014; Kisseleva and Brenner 2011), and our results indicated that TGF-β1-induced activation of LX-2 cells was accompanied with significant upregulation of both the mRNA and protein levels of GPR81. Consistent with the findings in vivo, treatment with the GPR81 agonist also enhanced TGF-β1-induced fibrogenesis in LX-2 cells. Although these results could not exclude that GPR81 on other cells also contributes to the induction of liver fibrosis, the present study suggests that GPR81 might be a crucial pathological factor that promotes the activation of HSCs and then, the development of liver fibrosis.

Previous studies have suggested that GPR81 is coupled to Gi, which suppresses adenylyl cyclase and leads to downregulation of cAMP, an essential intracellular secondary messenger required for the activation of PKA and its downstream target CREB (Ahmed et al. 2010; Zarneshan et al. 2022). CREB transcriptionally promotes the expression of anti-fibrotic genes, such as Smad7, a representative inhibitory Smad that contains CRE in its promotor (van Capelle 2020). Smad7 interacts with the receptor of TGF-β1 but it lacks the C-terminal SSXS motif that is required for the phosphorylation of Smad3 (Hu et al. 2018; Gao et al. 2022). Thus, Smad7 inhibits TGF-β1 signaling by competitively binding to the TGF-β1 receptor and blocking the phosphorylation of Smad3 (Tanner et al. 2023; Lee et al. 2022). In the present study, the inhibitory effects of DHBA/GPR81 on the anti-fibrotic CREB/Smad7 pathway have been verified in TGF-β1-activated LX-2 cells and CCl_4_-insulted mice, which might represent an important mechanism underlying the detrimental activity of GPR81 in liver fibrosis.

## Conclusion

**Fig. 7:**
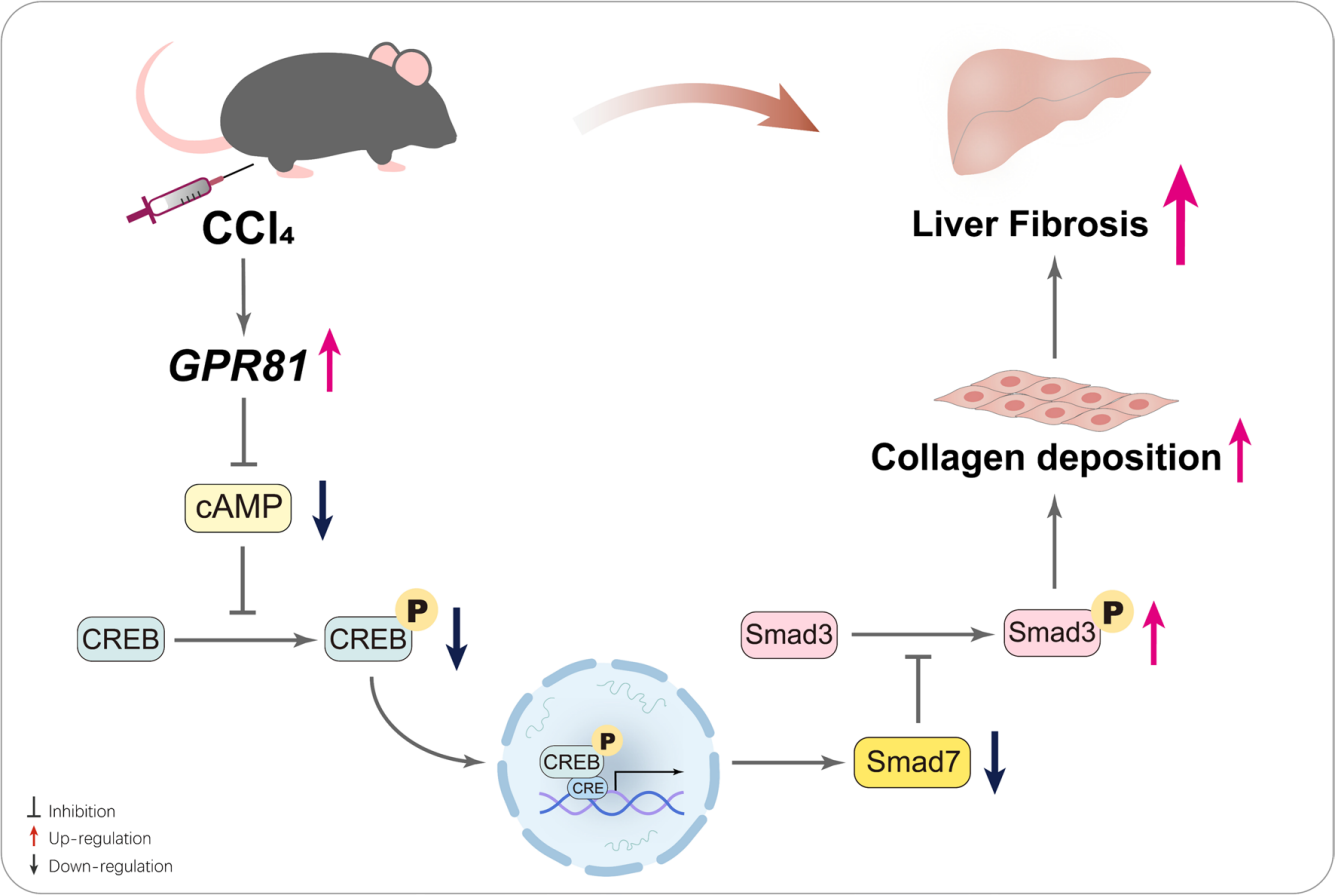
The schematic diagram of the mechanisms underlying the pro-fibrotic activities of GPR81. The development of liver fibrosis is associated with upregulation of GPR81. GPR81 is a Gi-coupled receptor that decreases the level of cAMP and suppresses the activation of CREB. The suppressed CREB might reduce the expression of Smad7, an inhibitory Smad that competitively inhibits the activation of Smad3. Smad3 is essential for the transcription of collagen I and other pro-fibrotic genes. The reduction of Smad7 might weaken the inhibition of Smad3, resulting in uncontrolled Smad3 signaling, enhanced collagen production, and aggravated liver fibrosis

Taken together, the present study found that liver fibrosis is associated with the upregulation of GPR81, which might be a detrimental event that promotes the development of liver fibrosis via suppression of CREB/Smad7 pathway (Fig. 7). Although the molecular mechanisms underlying the pathological roles of GPR81 in liver fibrosis remain to be further investigated, this is the first study that revealed the pathological significance of GPR81 in liver fibrosis.

### Supplementary Information

Suplementary Material 1.

Suplementary Material 2.

Suplementary Material 3.

Suplementary Material 4.

Suplementary Material 5.

Suplementary Material 6.

## Data Availability

Data sharing is not applicable to this article as no datasets were generated or analyzed during the current study.

## Abbreviations

ALT: Alanine aminotransferase
AST: Aspartate aminotransferase
α-SMA: Alpha smooth muscle actin
COL1A1: Collagen I
CCl_4_: Carbon tetrachloride
cAMP: Cyclic adenosine monophosphate
CREB: CAMP-responsive element-binding protein
DHBA: 3,5-Dihydroxybenzoic acid
GPR81: G-protein-coupled receptor 81
HCAR1: Hydroxycarboxylic acid receptor 1
HSCs: Hepatic stellate cells
HYP: Hydroxyproline
IL-6: Interleukin-6
KO: Knockout
PKA: Protein kinase A
TGF-β1: Transforming growth factor beta 1
TNF-α: Tumor necrosis factor alpha
WT: Wild type
