# Supplementary material for: Deletion of GPR81 activates CREB/Smad7 pathway and alleviates liver fibrosis in mice
Source: Mol Med. 2024 Jul 9;30:99. doi: 10.1186/s10020-024-00867-y (PMC11234765; doi:10.1186/s10020-024-00867-y)
Supplement: Supplementary file 1 — Suplementary Material 1. [file 10020_2024_867_MOESM1_ESM.docx]

**Supplementary methods**

**Determination of lactate level**

To evaluated the association between lactate and liver fibrosis/hepatic stellate cells (HSCs) activation, the levels of lactate in the serum from mice with CCl_4_-induced liver fibrosis and cell lysate from TGF-β1-activated hepatic stellate cell line LX-2 were determined with the Lactic Acid assay kit (Nanjing Jiancheng Biotech) according to the manufacturer’s instructions.

**Determine the effects of DHBA in GPR81 deficiency mice with CCl_4_ challenge**

To determine the potential effects of DHBA in CCl_4_-challenged GPR81 deficiency mice, GPR81 knockout (KO) mice with intraperitoneal injection of CCl_4_ (1 ml/kg, dissolved in olive oil, twice per week, for 8 weeks) were daily treated with GPR81 agonist DHBA (30 mg/kg, dissolved in normal saline) or the vehicle (saline). Three days after the last injection of CCl_4_, the mice were anesthetized with 5% chloral hydrate and sacrificed. The left lobe of the liver and the blood sample were harvested for further experiments.

**Determine the effects of lactate on the activation of LX-2 cells**

LX-2 cells were exposed to FBS-free medium overnight, and then the culture media was replaced by fresh media supplemented with recombinant human TGF-β1 (#100-21, PeproTech) at a concentration of 10 ng/ml or the vehicle. To determine the effects of lactate on LX-2 cells, lacate were supplemented at a concentration of 20mM. 24 hours later, cells were collected for mRNA or protein extraction.

**Supplementary figure legends**

**Supplementary Figure 1 Liver fibrosis and HSCs activation were associated with elevation of lactate.** (A) Mice were intraperitoneally injected with olive oil or CCl_4_ for 8 weeks to induce liver fibrosis. The serum lactate levels were examined and were expressed as a fold change relative to the vehicle group (n=4). (B) Human hepatic stellate cells (HSCs) LX-2 were treated with vehicle or TGF-β1 to induce HSCs activation. The intracellular lactate levels were detected and were expressed as a fold change relative to the vehicle group (n=4). All data were expressed as mean ± SD.

**Supplementary Figure 2** **Deletion of GPR81 alleviated CCl_4_-induced morphological abnormalities of liver.** WT mice or GPR81 KO mice were exposed for 8 weeks to CCl_4_ to induce liver fibrosis. Representative image of liver from each group was shown.

**Supplementary Figure 3** **GPR81 activator DHBA aggravated CCl_4_-induced chronic liver injury.** Mice with CCl_4_-induced liver fibrosis were supplemented with or without DHBA for 8 weeks. (A) Representative image of liver from each group was shown. (B)The serum ALT and AST levels were examined (n=4). (C) The mRNA level of TNF-α and IL-6 were detected (n=4). All data were expressed as mean ± SD.

**Supplementary Figure 4 Deletion of GPR81 abolished the profibrotic effects of DHBA.** GPR81 KO mice with CCl_4_-induced liver fibrosis were supplemented with DHBA for 8 weeks. (A) The serum ALT and AST levels were examined (n=4). (B) The mRNA level of TNF-α, IL-6, TGF-β1, α-SMA, and COL1A1 were detected (n=4). All data were expressed as mean ± SD.

**Supplementary Figure 5 Supplementation of lactate enhanced HSCs activation.** Hepatic stellate cells (HSCs) LX-2 with TGF-β1 exposure were supplemented with lactate for 24 h. (A) The mRNA expressions of COL1A1 were examined (n=4). (B) Relative protein expression of α-SMA and COL1A1 were examined (n=4). All data were expressed as mean ± SD.
