# Supplementary figures and images for: Deletion of GPR81 activates CREB/Smad7 pathway and alleviates liver fibrosis in mice
Source: Mol Med. 2024 Jul 9;30:99. doi: 10.1186/s10020-024-00867-y (PMC11234765; doi:10.1186/s10020-024-00867-y)

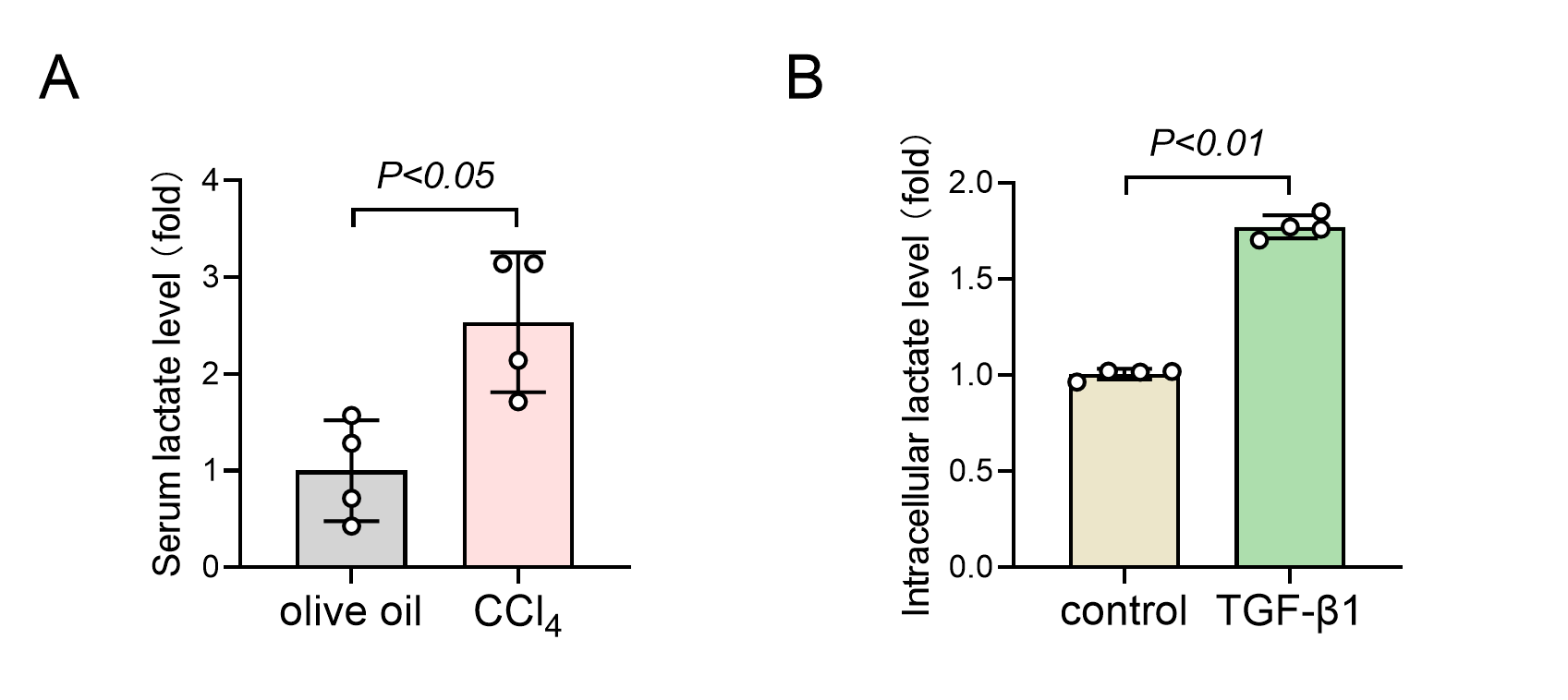

Supplement: Supplementary file 2 — Suplementary Material 2. [file 10020_2024_867_MOESM2_ESM.tif]

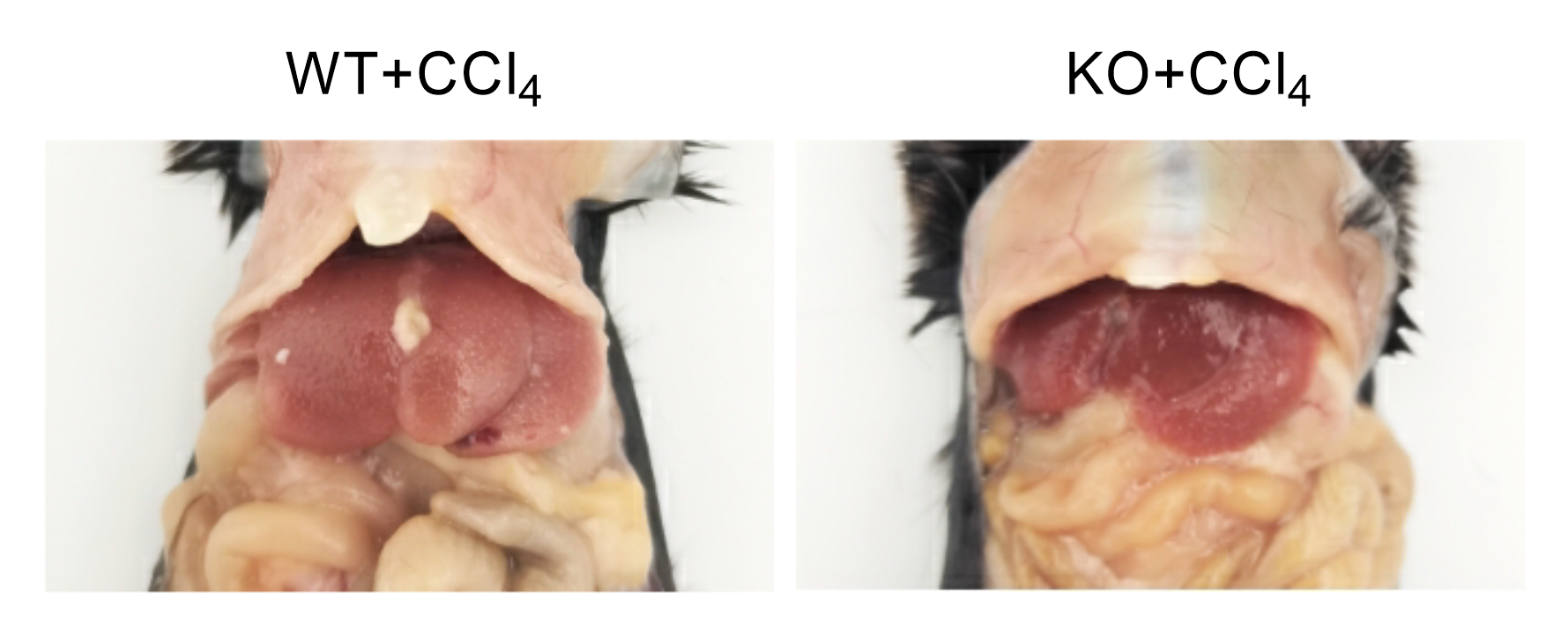

Supplement: Supplementary file 3 — Suplementary Material 3. [file 10020_2024_867_MOESM3_ESM.tif]

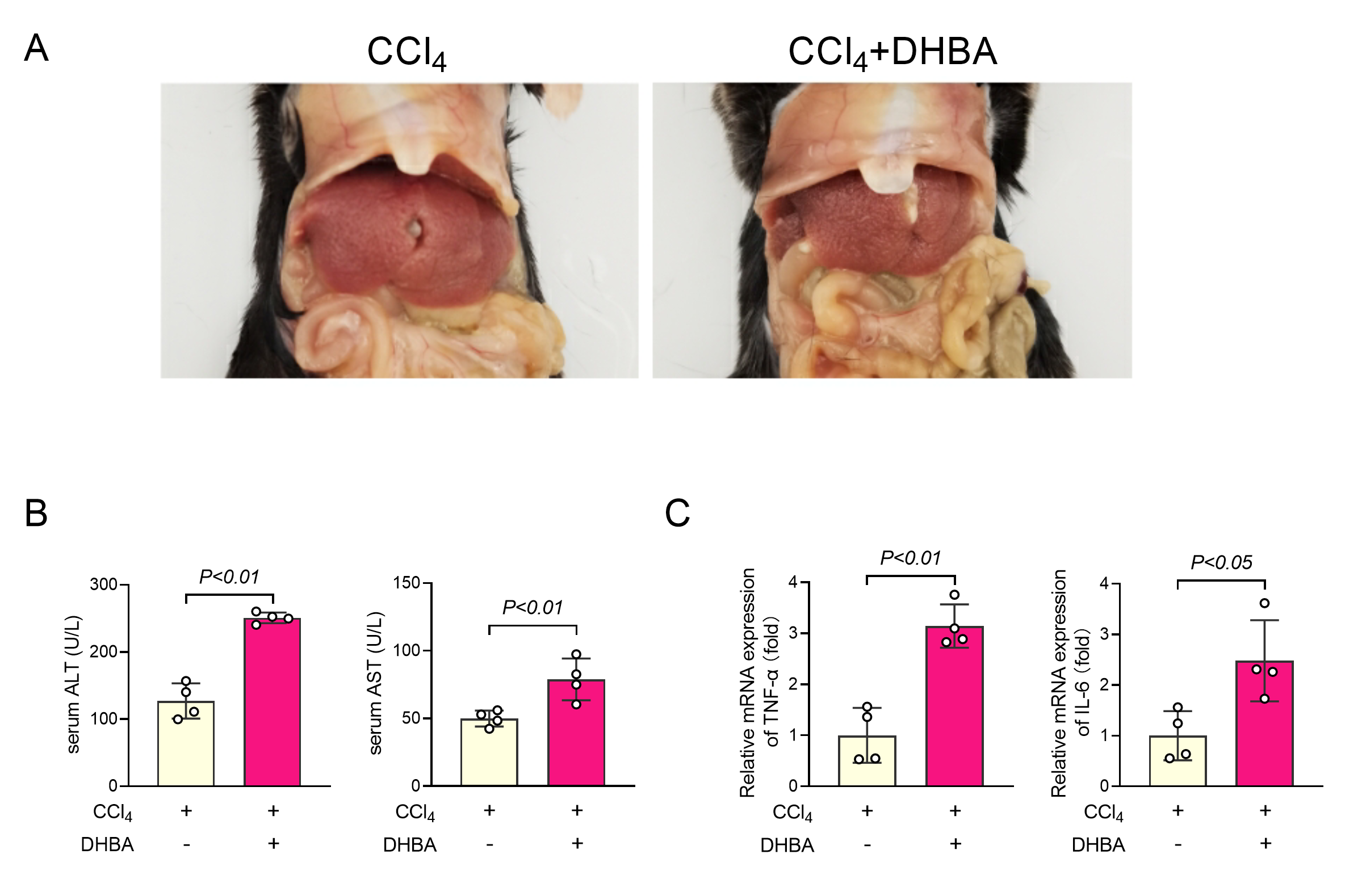

Supplement: Supplementary file 4 — Suplementary Material 4. [file 10020_2024_867_MOESM4_ESM.tif]

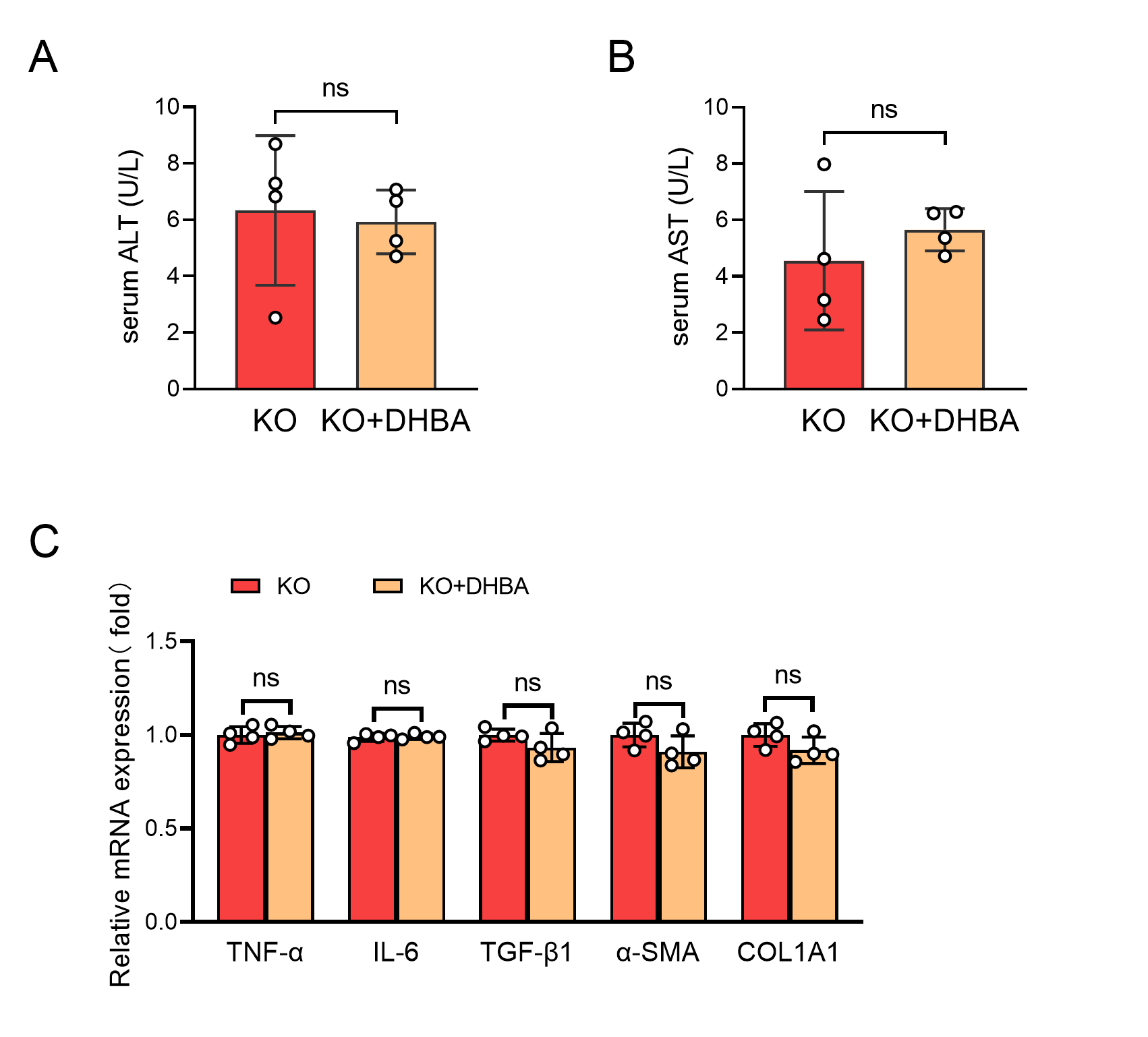

Supplement: Supplementary file 5 — Suplementary Material 5. [file 10020_2024_867_MOESM5_ESM.tif]

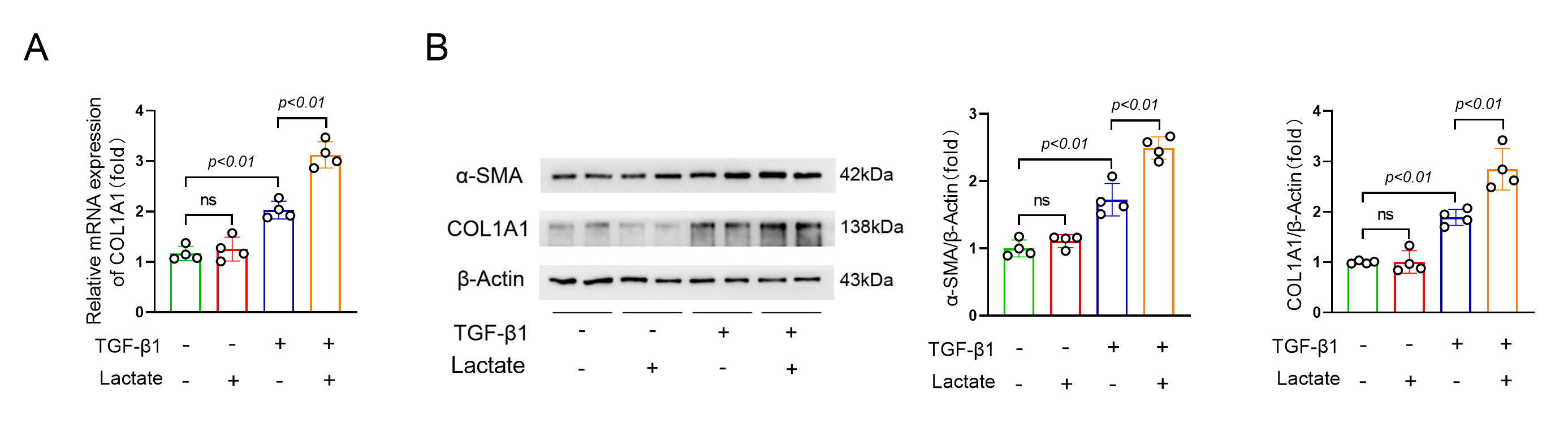

Supplement: Supplementary file 6 — Suplementary Material 6. [file 10020_2024_867_MOESM6_ESM.tif]
